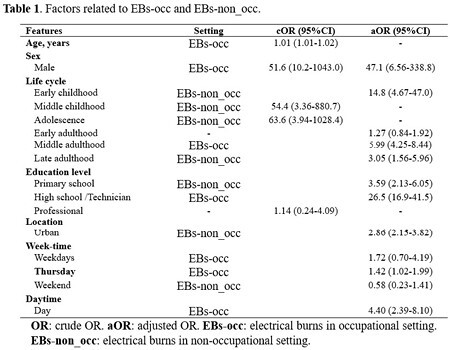# 738 Electrical Burn Injuries in Occupational and Non-occupational Settings from 2010 to 2021: Cross-sectional Design

**DOI:** 10.1093/jbcr/irae036.281

**Published:** 2024-04-17

**Authors:** Karen P Ayala, Aníbal A Teherán, Luis M Pombo, Ginna P Tocanchon, Carol A Zuluaga-Ortíz, Gabriel Camero-Ramos, Albert A Ávila

**Affiliations:** Fundación Universitaria Juan N Corpas, Bogota, Distrito Capital de Bogota; Cruz Roja Colombiana secional Cundinamarca, Bogotá, Bogotá, Distrito Capital de Bogota; Burns Critical Care Unit, Hospital Simón Bolivar, Bogotá, Distrito Capital de Bogota; Fundación Universitaria Juan N Corpas, Bogota, Distrito Capital de Bogota; Cruz Roja Colombiana secional Cundinamarca, Bogotá, Bogotá, Distrito Capital de Bogota; Burns Critical Care Unit, Hospital Simón Bolivar, Bogotá, Distrito Capital de Bogota; Fundación Universitaria Juan N Corpas, Bogota, Distrito Capital de Bogota; Cruz Roja Colombiana secional Cundinamarca, Bogotá, Bogotá, Distrito Capital de Bogota; Burns Critical Care Unit, Hospital Simón Bolivar, Bogotá, Distrito Capital de Bogota; Fundación Universitaria Juan N Corpas, Bogota, Distrito Capital de Bogota; Cruz Roja Colombiana secional Cundinamarca, Bogotá, Bogotá, Distrito Capital de Bogota; Burns Critical Care Unit, Hospital Simón Bolivar, Bogotá, Distrito Capital de Bogota; Fundación Universitaria Juan N Corpas, Bogota, Distrito Capital de Bogota; Cruz Roja Colombiana secional Cundinamarca, Bogotá, Bogotá, Distrito Capital de Bogota; Burns Critical Care Unit, Hospital Simón Bolivar, Bogotá, Distrito Capital de Bogota; Fundación Universitaria Juan N Corpas, Bogota, Distrito Capital de Bogota; Cruz Roja Colombiana secional Cundinamarca, Bogotá, Bogotá, Distrito Capital de Bogota; Burns Critical Care Unit, Hospital Simón Bolivar, Bogotá, Distrito Capital de Bogota; Fundación Universitaria Juan N Corpas, Bogota, Distrito Capital de Bogota; Cruz Roja Colombiana secional Cundinamarca, Bogotá, Bogotá, Distrito Capital de Bogota; Burns Critical Care Unit, Hospital Simón Bolivar, Bogotá, Distrito Capital de Bogota

## Abstract

**Introduction:**

Burns affect 11 million people worldwide annually. Electrically related burns are renowned for inflicting extensive harm and long-term consequences that can lead to severe illness and fatalities. In occupational and non-occupational settings people may be exposed to electrical burns (EBs), which can lead to functional or anatomical consequences. We identified sociodemographic features related to electrical burns in occupational (EBs-occ) and non-occupational (EBs-non_occ) settings.

**Methods:**

A cross sectional design, using an open dataset of electrical injuries occurred during 2010-2021 period. Sociodemographic features of people injured in EBs-occ and EBs-non_occ were described counts (%), Incidence*million people (I0;95%CI). To identify related factors (age-sex adjusted) with injuries in EBs-occ and EBs-non_occ, we applied a Binary Logistic Regression (aOR).

**Results:**

Over the course of 11 years occurred 1.274 EBs (I0: 2.47;2.34-2.61), 287 EBs-occ (I0: 1.35;1.20-1.51) and 987 EBs-non_occ (I0: 3.25;3.05-3.46). Age median was 31 years, most cases distributed in middle adulthood (52.8%), male (88.1%), and people with high school/technician (42.8%), urban location (73.7%), weekdays (95.3%), and during daytime hours (85.5%). Factors related to EBs-occ were male sex, middle adulthood, high school/technician, and occurred mainly on thursday and daytime hours. Otherwise, EBs-non_occ factors were early childhood, primary school, urban location, and occurred mostly on weekends.

**Conclusions:**

Both occupational and non-occupational settings pose a risk of injuries to individuals in the workplace. We have identified sociodemographic factors related to these injuries in both occupational and non-occupational settings, which could help to avoid damages and long-term complications, especially among vulnerable individuals such as those at an extreme age.

**Applicability of Research to Practice:**

This information could be helpful in refining existing preventive measures, particularly for non-occupational electrical burns.